# Supplementary material for: Neural timescales reflect behavioral demands in freely moving rhesus macaques
Source: Nat Commun. 2024 Mar 9;15:2151. doi: 10.1038/s41467-024-46488-1 (PMC10925022; doi:10.1038/s41467-024-46488-1)
Supplement: Supplementary file 1 — Supplementary Information [file 41467_2024_46488_MOESM1_ESM.pdf]

SUPPLEMENTARY MATERIALS:
Neural timescales reflect behavioral demands in freely moving rhesus macaques

Ana M.G. Manea[1,2], David J.-N. Maisson, Benjamin Voloh, Anna Zilverstand[3], Benjamin Hayden[4], Jan Zimmermann[1,2]

Affiliations
1 Department of Neuroscience, University of Minnesota, Minneapolis MN
2 Center for Magnetic Resonance Research, University of Minnesota, Minneapolis MN
3 Department of Psychiatry and Behavioral Sciences, University of Minnesota, Minneapolis MN
4 Department of Neurosurgery, Baylor College of Medicine, Houston, TX

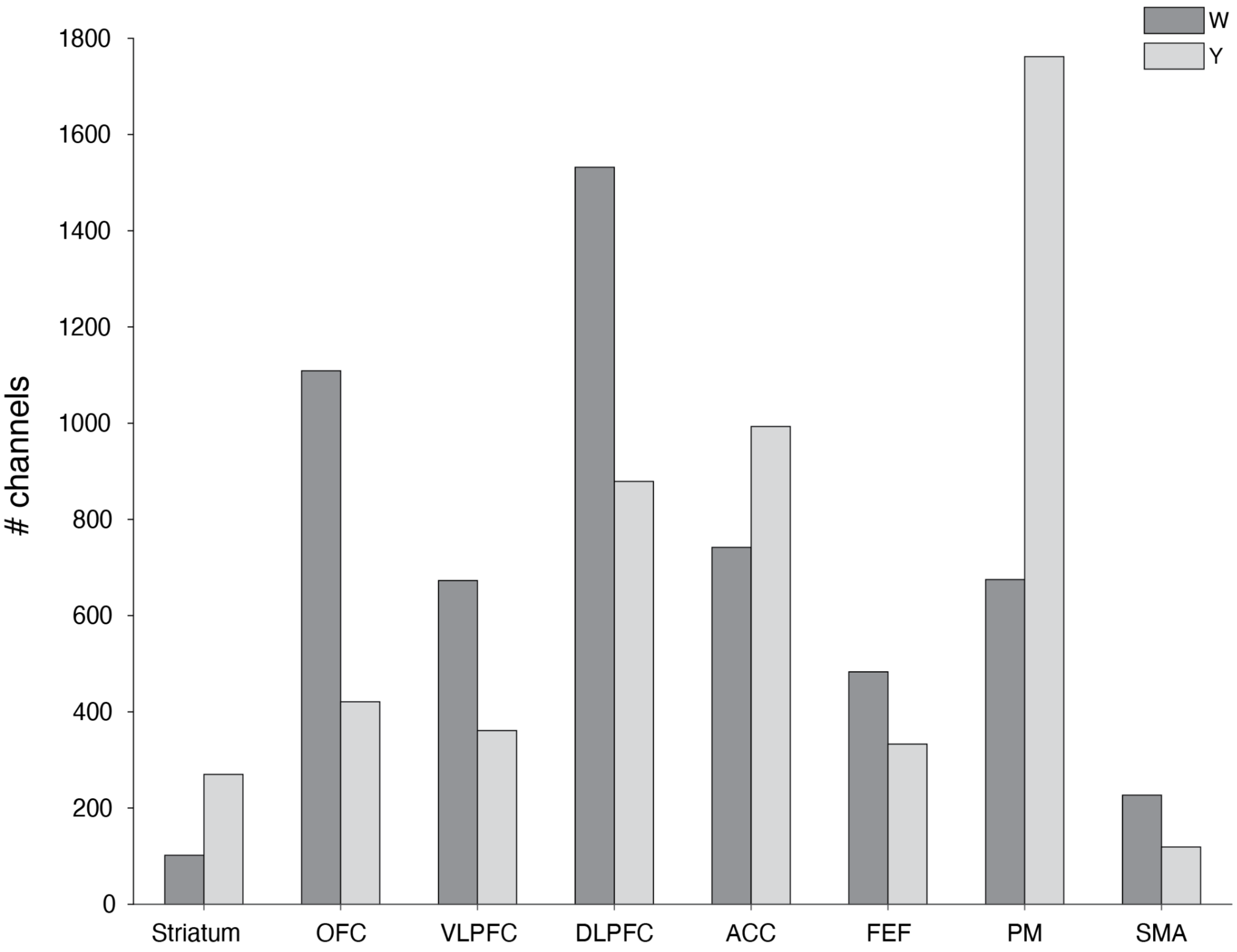

Supplementary Fig. 1 The number of channels per area for Monkey Y and Monkey W. Source data are provided as a Source Data file.

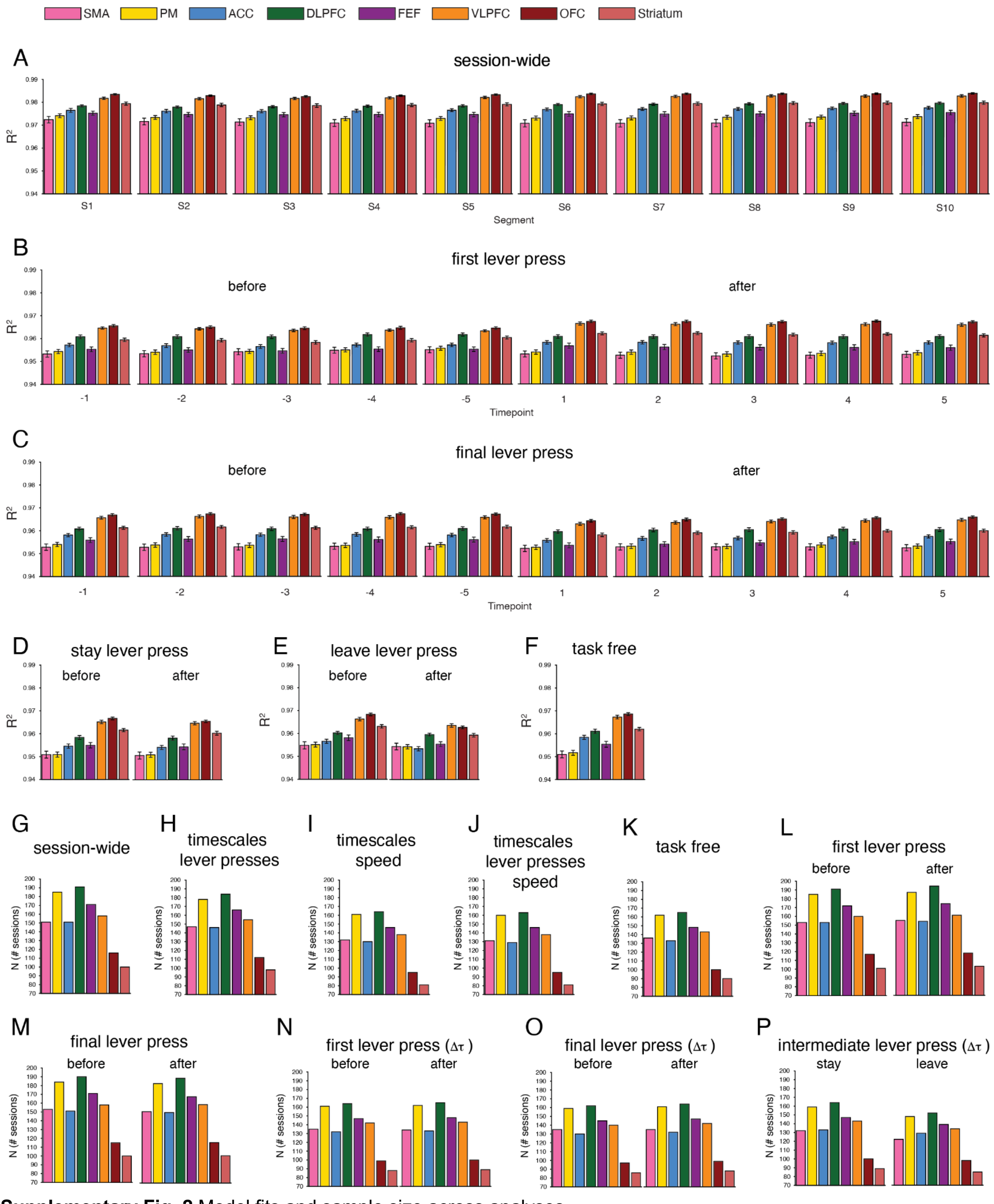

Supplementary Fig. 2 Model fits and sample size across analyses.

(A) The average model fits across sessions for individual areas in each 10-min time segment ± s.e.m. This panel depicts the model fits for the analysis in **Fig. 2A**. Y-axis: average R^2. X-axis: time segments. **(B)** The average model fits across sessions for individual areas before and after the first lever press ± s.e.m. This panel depicts the model fits for the analyses in **Fig. 3C**, **Fig. 4B** and **Fig. 5A**. Y-axis: average R^2. X-axis: time points before (-1: -5) and after (1:5) the lever press. **(C)** The average model fits across sessions for individual areas before and after the final lever presses ± s.e.m. This panel depicts the model fits for the analyses in **Fig. 3D**, **Fig. 4C** and **Fig. 5B**. Y-axis: average R^2. X-axis: time points before (-1: -5) and after (1:5) the lever presses. **(D)** The average model fits across sessions for individual areas before and after the stay lever presses ± s.e.m. This panel depicts the model fits for the analysis in **Fig. 5C**. Y-axis: average R^2. X-axis: time points before (-1) and after (1) the lever press. **(E)** The average model fits across sessions for individual areas before and after the leave lever presses ± s.e.m. This panel depicts the model fits for the analysis in Fig. 5D. Y-axis: average R^2. X-axis: time points before (-1) and after (1) the lever press. **(F)** The average model fits across sessions for individual areas for the task free time

segments ± s.e.m. This panel depicts the model fits for the analysis in F**ig. 3B**. Y-axis: average R^2. **(G)** The number of sessions used to estimate the session-wide neural timescales in **Fig. 2A**. (**H)** The number of sessions (i.e., the sample size) for the neural timescales-task engagement analysis in **Fig. 2C**. (**I)** The number of sessions (i.e., the sample size) for the neural timescales-speed of movement analysis in **Fig. 2D**. (**J)** The number of sessions (i.e., the sample size) for the regression analysis in **Fig. 2E** and **Supplementary Fig. 3**. (**K)** The number of sessions (i.e., the sample size) for the task free neural timescales in **Fig. 3B**. (**L)** The number of sessions (i.e., the sample size) for the first lever presses analyses in **Fig. 3C** and **Supplementary Fig. 4A**. (**M)** The number of sessions (i.e., the sample size) for the final lever presses analyses in Fig. 3D and **Supplementary Fig. 4B**. (**N)** The number of sessions (i.e., the sample size) for the change in neural timescales from baseline for the first lever presses in **Fig. 4B**. (**O)** The number of sessions (i.e., the sample size) for the change in neural timescales from baseline analysis for the final lever presses in **Fig. 4C**. **(P)** The number of sessions (i.e., the sample size) for the change in neural timescales from baseline analysis for the intermediate lever presses in **Fig. 5C** (stay lever presses) and **Fig. 5D** (leave lever presses). Source data are provided as a Source Data file.

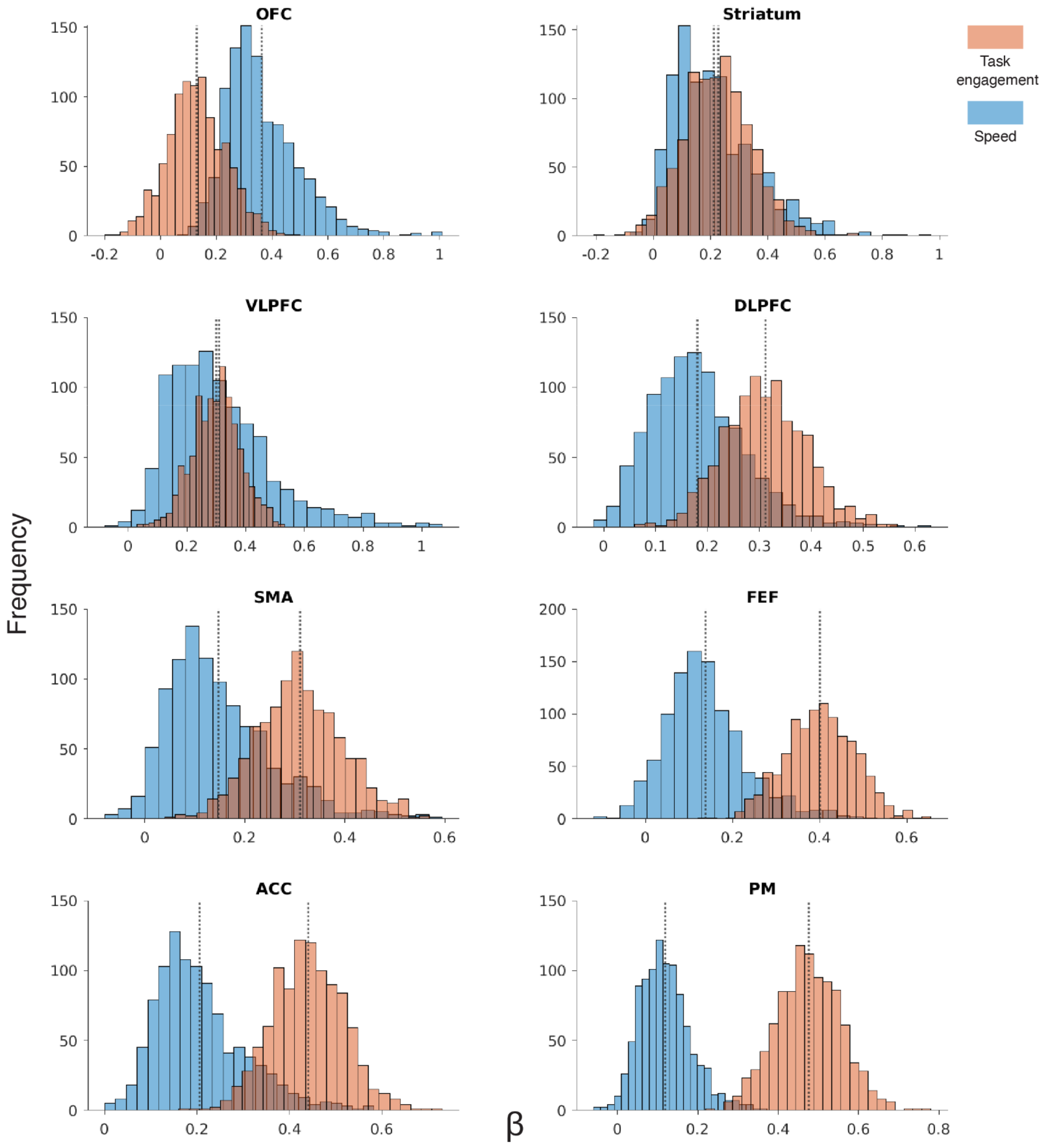

Supplementary Fig. 3 The distribution of standardized regression coefficients for task engagement and speed of movement.
To assess the relationship between task engagement, speed, and neural timescales, we employed a linear regression model with task engagement and speed as predictors and neural timescales as the dependent variable. For each area, we randomly sampled without replacement n (i.e., equivalent to the number of sessions) observations out of the total number of data points (note: the total number of data points per area can be calculated as the number of sessions x 10, i.e., number of time segments; see Supplementary Fig. 2J for the sample size of this analysis, as reflected by the number of recording sessions per area). For each subsample, we fit a linear regression model. Dotted line: the mean of the distributions. Source data are provided as a Source Data file.

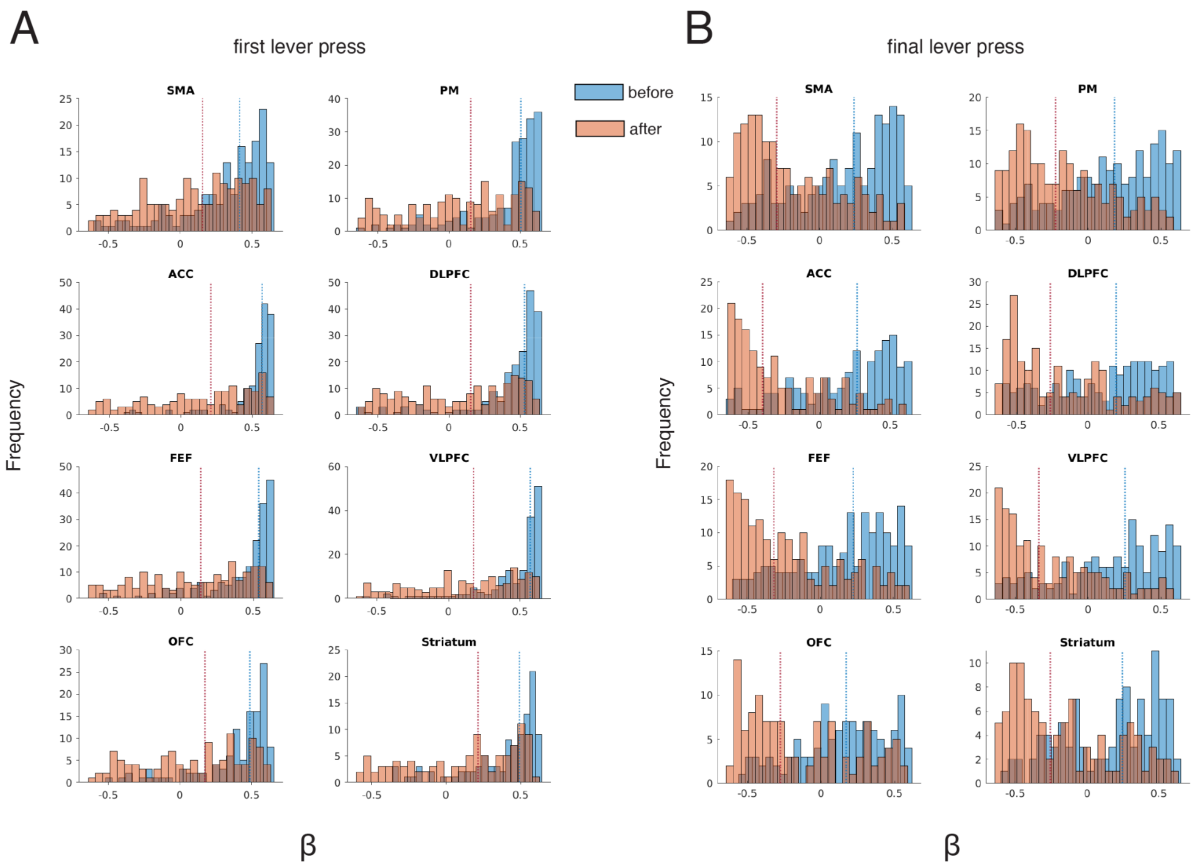

Supplementary Fig. 4 The distribution of standardized regression coefficients.

(A) The distribution of standardized regression coefficients for the first lever press. The average R^2 per area across sessions for: (1) **before**: 0.47 ± 0.03 (SMA); 0.64 ± 0.02 (PM); 0.81 ± 0.02 (ACC); 0.71 ± 0.02 (DLPFC); 0.72 ± 0.02 (FEF); 0.81 ± 0.02 (VLPFC); 0.61 ± 0.03 (OFC); 0.60 ± 0.03 (Striatum); (2) **after**: 0.21 ± 0.02 (SMA); 0.23 ± 0.02 (PM); 0.3 ± 0.03 (ACC); 0.34 ± 0.02 (DLPFC); 0.29 ± 0.02 (FEF); 0.28 ± 0.02 (VLPFC); 0.28 ± 0.03 (OFC); 0.31 ± 0.03 (Striatum). Dotted line: the median of the distributions. (B) The distribution of standardized regression coefficients for the final lever press. The average R^2 per area across sessions for: (1) **before**: 0.32 ± 0.02 (SMA); 0.27 ± 0.02 (PM); 0.37 ± 0.03 (ACC); 0.3 ± 0.02 (DLPFC); 0.26 ± 0.02 (FEF); 0.29 ± 0.02 (VLPFC); 0.18 ± 0.03 (OFC); 0.25 ± 0.03 (Striatum); (2) **after**: 0.3 ± 0.02 (SMA); 0.29 ± 0.02 (PM); 0.46 ± 0.03 (ACC); 0.40 ± 0.02 (DLPFC); 0.38 ± 0.02 (FEF); 0.44 ± 0.03 (VLPFC); 0.35 ± 0.03 (OFC); 0.34 ± 0.03 (Striatum). Dotted line: the median of the distributions. Source data are provided as a Source Data file.

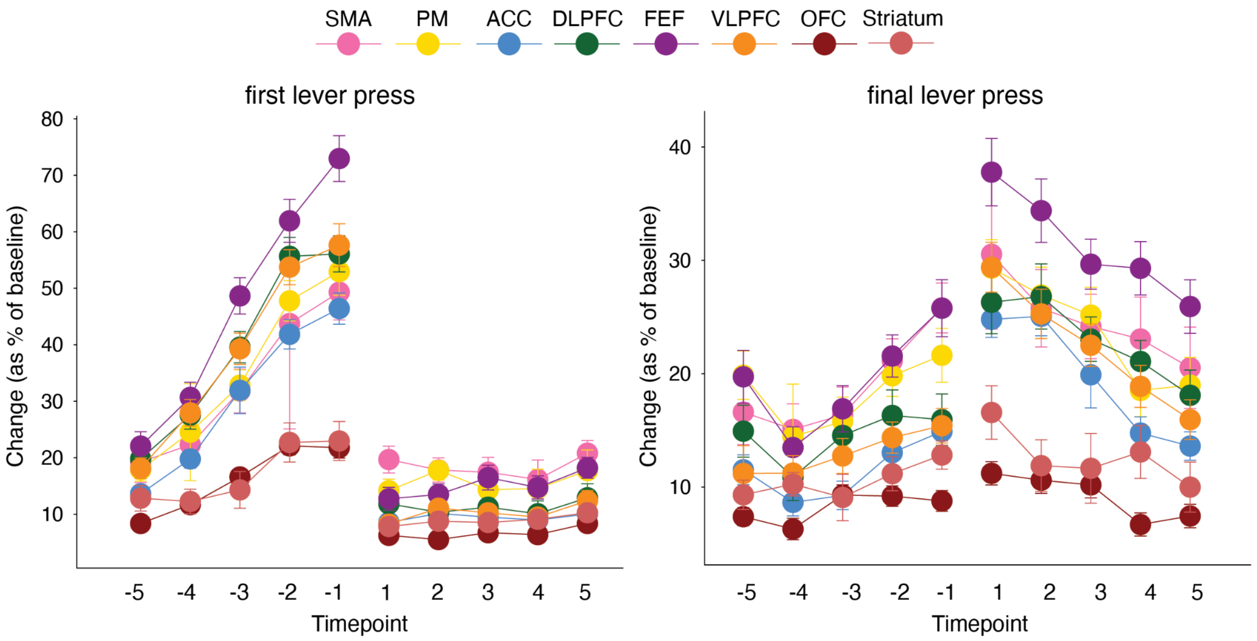

Supplementary Fig. 5. Area-specific adaptation of neural timescales represented as percentage of baseline. The change was calculated as the difference between event-related neural timescales (first and final lever presses) and resting-baseline divided by the resting-baseline. Circles: median across sessions ± s.e.m. Source data are provided as a Source Data file.

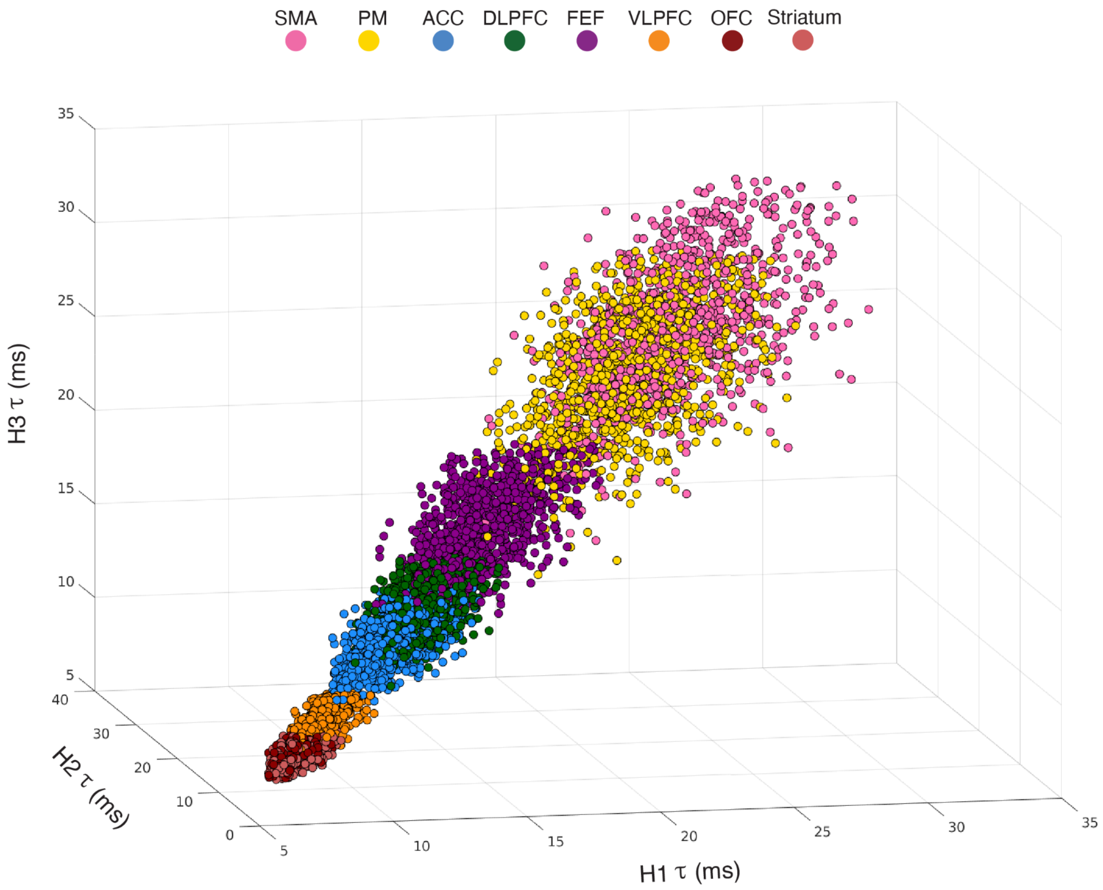

Supplementary Fig. 6 Session-wide neural timescales using different Hamming window sizes.
Neural timescales were estimated for 20 sessions (Monkey W: 10, Monkey Y: 10) based on PSDs estimated using 500 ms (H1, 250 ms overlap), 1000 ms (H2, 500 ms overlap), and 1500 ms (H3, 750 ms overlap) Hamming windows. The resulting neural timescales were collapsed across sessions and subjects by taking the median. For outlier removal, the timepoints with values larger than the 95th percentile were removed for H1, H2 and H3 to preserve the integrity of the timeseries. Irrespective of the Hamming window size, the relative position in the hierarchy is maintained. Circles: median neural timescales across sessions. Source data are provided as a Source Data file.

		SMA	PM	ACC	DLPFC	FEF	VLPFC	OFC	Striatum
Session wide	Mean	2.60	2.74	1.01	1.44	1.57	0.33	0.19	0.33
	SE	0.26	0.20	0.08	0.18	0.18	0.05	0.04	0.06
Task free	Mean	5.34	5.23	2.17	1.78	2.85	0.58	0.49	3.53
	SE	1.01	0.72	0.56	0.69	0.87	0.40	0.54	1.61
First lever press (before)	Mean	12.44	12.72	5.10	5.26	7.41	1.65	0.73	4.59
	SE	1.15	0.86	0.53	0.60	0.78	0.49	0.49	1.43
First lever press (after)	Mean	7.54	8.06	2.95	2.94	3.99	1.01	0.64	4.08
	SE	0.90	0.72	0.52	0.55	0.71	0.45	0.52	1.49
Final lever press (before)	Mean	8.35	8.82	3.38	3.46	4.67	1.14	0.64	4.22
	SE	0.94	0.72	0.52	0.59	0.72	0.46	0.52	1.51
Final lever press (after)	Mean	10.12	10.69	4.26	4.46	6.03	1.34	0.68	4.34
	SE	1.05	0.77	0.52	0.57	0.73	0.43	0.49	1.45
Stay lever press (before)	Mean	6.88	7.55	2.75	2.77	3.58	0.95	0.57	3.93
	SE	0.94	0.72	0.54	0.56	0.70	0.43	0.51	1.50
Stay lever press (after)	Mean	9.20	9.69	3.72	3.95	5.04	1.18	0.61	3.95
	SE	1.01	0.77	0.50	0.60	0.70	0.43	0.48	1.34
Leave lever press (before)	Mean	6.05	7.63	2.58	1.86	2.89	0.89	0.61	4.05
	SE	0.93	0.94	0.54	0.37	0.65	0.48	0.53	1.49
Leave lever press (after)	Mean	9.7	10.9	4.4	3.3	5.9	1.5	0.7	4.5
	SE	1.2	0.9	0.6	0.5	0.8	0.6	0.6	1.5

Supplementary Table 1 The percentage of discarded models across analyses.
The average percentage of discarded models across sessions and monkeys ± s.e.m. The percentage of discarded models was calculated for each session and channel as the total number of fitted models divided by the number of excluded models, and subsequently averaged across sessions and monkeys. The percentage of discarded models was independently calculated for each analysis: session-wide neural timescales (associated with **Fig. 2**), task free timescales (associated with **Fig. 3**), before and after the first and final lever presses (associated with **Fig. 3, Fig. 4,** and **Fig. 5**) before and after the stay and leave lever presses (associated with **Fig. 5**).
